# An imbalance between specialized pro-resolving lipid mediators and pro-inflammatory leukotrienes promotes instability of atherosclerotic plaques

**DOI:** 10.1038/ncomms12859

**Published:** 2016-09-23

**Authors:** Gabrielle Fredman, Jason Hellmann, Jonathan D. Proto, George Kuriakose, Romain A. Colas, Bernhard Dorweiler, E. Sander Connolly, Robert Solomon, David M. Jones, Eric J. Heyer, Matthew Spite, Ira Tabas

**Affiliations:** 1 Department of Anesthesiology, Perioperative and Pain Medicine, Departments of Medicine, Pathology & Cell Biology, and Physiology, Columbia University Medical Center, 630 West 168th Street, New York, New York 10032, USA; 2 The Department of Molecular and Cellular Physiology, Center for Cardiovascular Sciences, Albany Medical College, 47 New Scotland Avenue, Albany, New York 12208, USA; 3 Department of Anesthesiology, Perioperative and Pain Medicine, The Center for Experimental Therapeutics and Reperfusion Injury, Brigham and Women's Hospital and Harvard Medical School, Boston, Massachusetts 02115, USA; 4 Division of Vascular Surgery, Department of Cardiothoracic and Vascular Surgery, University Medical Center of the Johannes Gutenberg University, Langenbeckstraße 1, Mainz D-55131, Germany; 5 Department of Neurosurgery, Columbia University Medical Center, New York, New York 10032, USA; 6 The Department of Pathology, Albany Medical College, 47 New Scotland Avenue, Albany, New York 12208, USA; 7 Department of Anesthesiology, Columbia University Medical Center, New York, New York 10032, USA

## Abstract

Chronic unresolved inflammation plays a causal role in the development of advanced atherosclerosis, but the mechanisms that prevent resolution in atherosclerosis remain unclear. Here, we use targeted mass spectrometry to identify specialized pro-resolving lipid mediators (SPM) in histologically-defined stable and vulnerable regions of human carotid atherosclerotic plaques. The levels of SPMs, particularly resolvin D1 (RvD1), and the ratio of SPMs to pro-inflammatory leukotriene B_4_ (LTB_4_), are significantly decreased in the vulnerable regions. SPMs are also decreased in advanced plaques of fat-fed *Ldlr*^*−/−*^ mice. Administration of RvD1 to these mice during plaque progression restores the RvD1:LTB_4_ ratio to that of less advanced lesions and promotes plaque stability, including decreased lesional oxidative stress and necrosis, improved lesional efferocytosis, and thicker fibrous caps. These findings provide molecular support for the concept that defective inflammation resolution contributes to the formation of clinically dangerous plaques and offer a mechanistic rationale for SPM therapy to promote plaque stability.

## Abstract (web summary)

Atherosclerosis progression is linked to inflammatory processes in the blood vessel wall. Here, the authors show that, with the progression of atherosclerosis, the resolution of inflammation is impaired as the result of an imbalance between specialized pro-resolving lipid mediators and leukotrienes.

## Graphical abstract

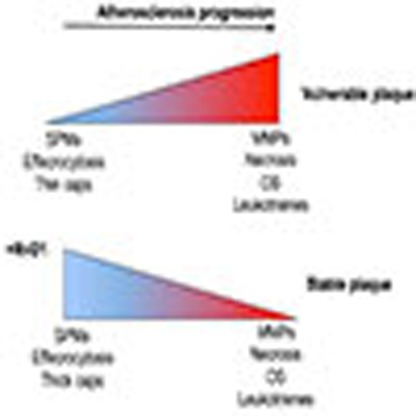

Chronic unresolved inflammation contributes to the development of advanced atherosclerosis^1^,^2^,^3^,^4^. However, the mechanisms that prevent resolution of inflammation in atherosclerosis are poorly understood. Specialized pro-resolving mediators (SPMs) represent a key family of resolution effectors that include arachidonic acid (AA)-derived lipoxins like LXA_4_ and docosahexaenoic acid (DHA)-derived resolvins like RvD1 (ref. 5). SPMs are autacoids, which by definition suggests that local tissue microenvironments play important roles in the production and function of these mediators^5^. In this regard, the balance of pro-inflammatory mediators and SPMs during acute inflammation regulates the duration of the inflammatory response and the timing of tissue resolution, for example, by promoting apoptosis and efferocytic clearance of inflammatory cells and other processes that dampen inflammation and repair collateral damage^6^,^7^. An imbalance between these two classes of mediators has been linked to a number of diverse chronic inflammatory diseases^8^,^9^,^10^.

In the case of atherosclerosis, major gaps remain in our understanding of the mechanisms and relevance of defective resolution. In humans, the subset of atherosclerotic plaques that are most important are those that at risk for precipitating acute atherothrombotic clinical events, including myocardial infarction, unstable angina, sudden cardiac death and stroke^11^. These so-called ‘vulnerable' plaques have distinct features, including heightened inflammation and oxidative stress (OS); prominent areas of necrosis, which are composed of dead cells not efficiently cleared by efferocytosis; and thinning of a protective layer of collagen (‘fibrous cap') that overlies the areas of necrosis in more stable lesions^2^,^11^,^12^. These features are suggestive of defective resolution, but the mechanisms of this defect in advanced atherosclerosis, including whether a deficiency of SPMs is involved, are not known.

A possible clue to mechanism comes from a previous study showing that macrophages in advanced atherosclerotic plaques have abundant nuclear 5-lipoxygenase (5-LOX)^13^. The potential significance of this observation is that nuclear 5-LOX, because of its proximity to LTA_4_ hydrolase, leads to the conversion of AA to pro-inflammatory/pro-atherogenic leukotrienes in macrophages^14^,^15^. In contrast, non-nuclear 5-LOX, potentially because of its proximity to 12/15-LOX, promotes the conversion of AA to pro-resolving LXA_4_ or DHA to RvD1 (ref. 16). Moreover, nuclear 5-LOX and the resultant decrease in SPMs might trigger a ‘negative SPM feedback' cycle, because we recently showed that RvD1 promotes nuclear exclusion of 5-LOX, which suppresses LTB_4_ and enhances LXA_4_ in macrophages^16^. Thus, a decrease in SPMs may beget a further decrease in SPM production by macrophages. However, the critical issue as to whether SPMs and the SPM:leukotriene balance are perturbed in advanced plaques is not known.

Here, we report that there is a significant imbalance between 5-LOX-derived SPMs, particularly RvD1, and leukotrienes, in human vulnerable plaques compared with stable lesions. Similar results were found in advanced murine atherosclerotic plaques, and in this model we show that restoration of lesional RvD1 suppresses plaque progression. These findings provide critical molecular support for the hypothesis that defective resolution contributes to plaque progression and for the concept that SPM ‘restoration' therapy could promote plaque stabilization.

## Results

### SPM and leukotriene imbalance in human vulnerable plaques

**Figure 1:**
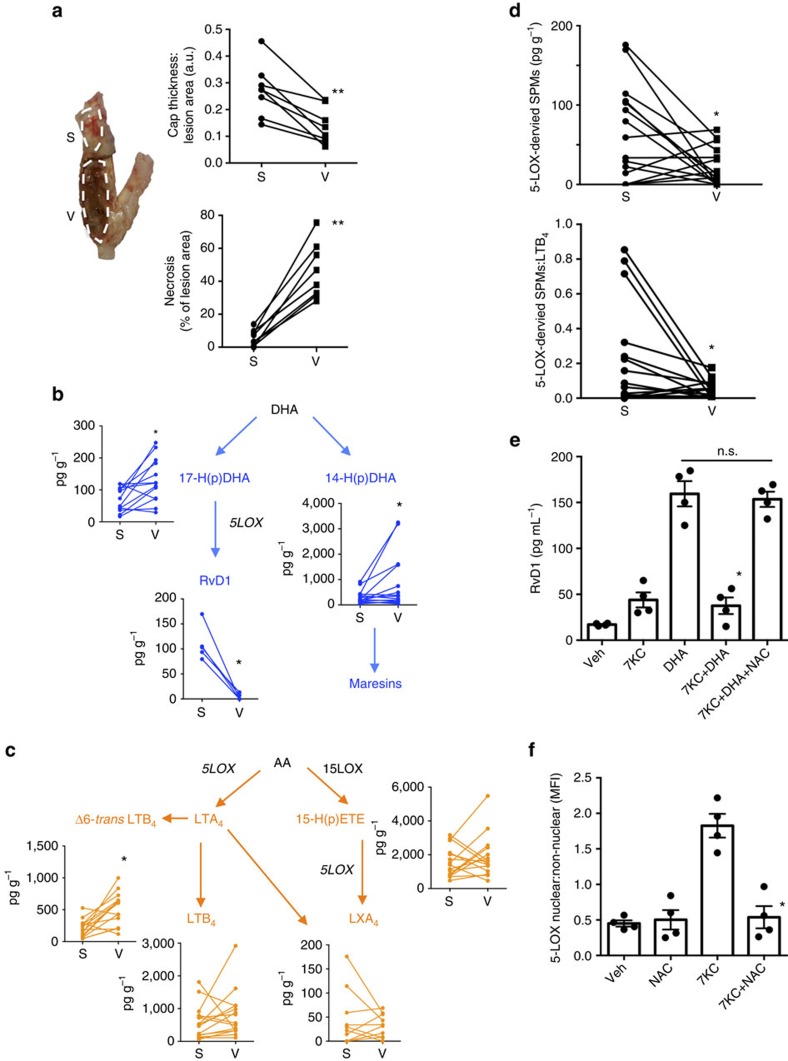
Human vulnerable atherosclerotic plaques have decreased 5-LOX SPM levels and a lower SPM:LT ratio than stable lesions. (**a**) Flash-frozen human plaques were separated into vulnerable (V) and stable (S) regions (left panel), which were quantified for fibrous cap thickness:lesion area ratio (top right panel) and percent plaque necrosis (bottom right panel). (*t*-test, **P<0.01, *n*=8 donors). (**b**) Quantification of key DHA-derived lipid mediators, with their respective biosynthetic pathways indicated (LOX, lipoxygenase). (**c**) Quantification of key AA-derived lipid mediators. (**d**) Comparison of 5-LOX-derived SPMs (RvD1 and LXA_4_) and the SPM:LT ratio in vulnerable versus stable regions. For (**b**–**d**), *t*-test, *P<0.05, *n*=15 donors. (**e**) Quantification of RvD1 by ELISA in human macrophages that were incubated for 5 h with vehicle control, or 35 μM of 7KC, followed by Veh or 10 μM DHA, for an additional 40 min, and then the media were assayed for RvD1 with or without a 1 h pre-incubation with 10 μM NAC. Data are shown as mean±s.e.m. (*n*=4 separate donors). Statistical analysis was conducted using one-way ANOVA with the Kruskal–Wallis test and Dunn's multiple comparison *post-hoc* analysis, *P<0.05 of *n*=4 separate donors. (**f**) Quantification nuclear:non-nuclear 5-LOX ratio by confocal microscopy in macrophages incubated as in **e**, with two additional groups, NAC alone and 7KC+NAC. Images were acquired on a Leica confocal microscope, and nuclear:non-nuclear 5-LOX MFI was quantified using Image J. Data are shown as mean±s.e.m. (*n*=4 separate donors). Statistical analysis was conducted using one-way ANOVA with the Kruskal–Wallis test with the Dunn's multiple comparison *post-hoc* analysis, *P<0.05.

Flash-frozen human plaques obtained from carotid endarterectomy were dissected into ‘vulnerable' and ‘stable' regions based on gross appearance (Fig. 1a left panel) and quantification of features of plaque vulnerability, such as fibrous cap thickness, plaque necrosis, and OS (Fig. 1a right top and bottom panels, Supplementary Fig. 1a) was carried out^17^,^18^. Fibrous caps and necrosis were assessed by histology, and OS was quantified by mean fluorescence intensity after staining with dihydroethidium (DHE), a fluorescent probe that detects superoxide in tissues. Fibrous caps were significantly thicker in the regions that were assigned to the stable group versus vulnerable group (Fig. 1a, top right). Necrosis and DHE were higher in the regions that were assigned to the vulnerable group versus stable group (Fig. 1a bottom right panel and Supplementary Fig. 1a). Patient characteristics are shown in Supplementary Table 1.

The vulnerable and stable regions were subjected to liquid chromatography-tandem mass spectrometry (LC–MS/MS) for targeted lipid mediator analysis. The major SPMs detected were those that require 5-LOX for biosynthesis, namely, LXA_4_ and RvD1, as depicted in the DHA and AA metabolomes in Fig. 1b,c. The values are represented as pg of lipid mediators per gram of tissue; the average weights of the specimens are shown in Supplementary Table 2. Whereas the resolvin and maresin biosynthetic intermediates 17- and 14-HDHA were significantly higher in vulnerable compared with stable regions, RvD1 was markedly decreased in the vulnerable regions. Interestingly, there was a significant correlation between RvD1 and DHE levels (Supplementary Fig. 1b), in which increased plaque reactive oxygen species (ROS) correlated with decreased RvD1 levels. In terms of AA-derived products, vulnerable regions contained higher levels of 6-*trans* LTB_4_ (*P*<0.05, *t*-test), the non-enzymatic hydrolysis product of LTA_4_, and there was a trend for lower levels of LXA_4_ (Fig. 1c). When the two regions were compared for total 5-LOX-derived SPMs and for the ratio of these SPMs to LTB_4_, vulnerable regions showed a striking decrease in both parameters (Fig. 1d). To further interrogate these findings, we compared vulnerable and stable regions from clinically defined symptomatic or asymptomatic plaques for the above parameters. Total 5-LOX-derived SPMs and the ratio of these SPMs to LTB_4_ was lower in the vulnerable regions compared with stable areas of the symptomatic plaques (Supplementary Fig. 1c), whereas these relationships between lipid mediators and plaque region were not statistically significant in the asymptomatic plaques (Supplementary Fig. 1d). The full MS/MS spectra of RvD1, LXA_4_, LTB_4_ and LXB_4_ identified in lesions are shown in Supplementary Fig. 2a. This biochemical signature of inflammation-resolution imbalance has previously been linked to other human inflammatory diseases^8^,^9^,^10^. We also identified other SPMs in lesions, including protectin D1 (PD1), 17*R*-RvD3 and LXB_4_; the levels of these SPMs, as well as the COX products prostaglandins E_2_ and D_2_, were not different in vulnerable versus stable plaques (Supplementary Fig. 2b,c; Tables 1 and 2). RvE1 and maresin 1 (MaR1) were not detected in these samples (Tables 1 and 2). In contrast, 5-HEPE and 15-HEPE, which are 5-LOX and 15-LOX monohydroxy products of EPA, respectively, were increased in vulnerable regions, with a trend in that direction for COX-2-derived 18-HEPE (Supplementary Fig. 2d), suggesting that 5-LOX, 15-LOX and COX-2 are active in vulnerable plaques. Thus, 5-LOX-derived SPMs are much lower in vulnerable versus stable regions of human atherosclerotic plaques despite the fact that the cells in the vulnerable regions are still bioactive.

### 7-KC-induced OS suppresses RvD1 production

Because vulnerable plaque regions had significantly more OS and significantly less RvD1, we next asked whether OS could impair RvD1 production as a possible mechanism of the decrease in SPMs in vulnerable plaques. We incubated human macrophages with a known inducer of OS found in human plaques, 7-ketocholesterol (7KC)^19^,^20^, and confirmed that 7KC stimulated OS in a manner that was suppressed by the anti-oxidant N-acetylcysteine (NAC) (Supplementary Fig. 3a). Under the conditions of this experiment, 7KC did not induce apoptosis in the macrophages, as indicated by no increase in cleaved caspase-3, which is a measure of caspase-3 activation (Supplementary Fig. 3b). Using this model, we found that DHA-stimulated RvD1 production by human macrophages was suppressed by 7KC and that this suppression was abrogated by co-treatment with NAC (Fig. 1e). As one possible mechanism for this effect, we considered that 7KC may increase the ratio of nuclear:non-nuclear 5-LOX, which would decrease the production of RvD1 (ref. 16). Indeed, 7KC increased this ratio in a manner that was abrogated by NAC (Fig. 1f).

To further probe mechanism, we considered our previous finding that CaMKII promotes nuclear 5-LOX by activating MAPKAPK2 (MK2)-mediated phosphorylation of Ser 271 on 5-LOX^16^. In view of the data presented in the previous paragraph, we were particularly interested in the finding by Anderson and colleagues^21^,^22^ that, under OS conditions, oxidation of paired methionine residues in the regulatory domain of CaMKII (oxCaMKII) sustains the enzyme's kinase activity in the absence of Ca^2+^/calmodulin. In this regard, we found that 7KC increases oxCaMKII in human macrophages, and this increase was abrogated by RvD1 (Supplementary Fig. 3c). We also found that 7KC led to the phosphorylation of both MK2 and 5-LOX-Ser271 and that RvD1 significantly reduced both of these endpoints (Supplementary Fig. 3d,e). To prove causation, we showed that silencing of CaMKII in human macrophages suppressed the ability of 7KC to stimulate 5-LOX nuclear localization (Supplementary Fig. 3f,g). In addition, the ability of RvD1 to decrease 7KC nuclear localization was not enhanced in CaMKII-silenced cells, suggesting that RvD1 exerts its action in a CaMKII-dependent manner. Lastly, we found that CaMKII silencing prevented the 7KC-mediated decrease of conversion of DHA to RvD1 (Supplementary Fig. 3h). Note that 7KC stimulation did not upregulate eicosanoid oxidoreductase, an enzyme known to metabolize RvD1, which is consistent with concept that the decrease in RvD1 by 7KC is primarily due to a production defect rather than enzymatic inactivation (Supplementary Fig. 4a). In summary, one mechanism of decreased SPMs in advanced lesions could be decreased SPM synthesis by macrophages via ROS-mediated activation of the CaMKII-nuclear 5-LOX pathway.

### Atheroprogression is associated with a decrease in lesional RvD1

**Figure 2:**
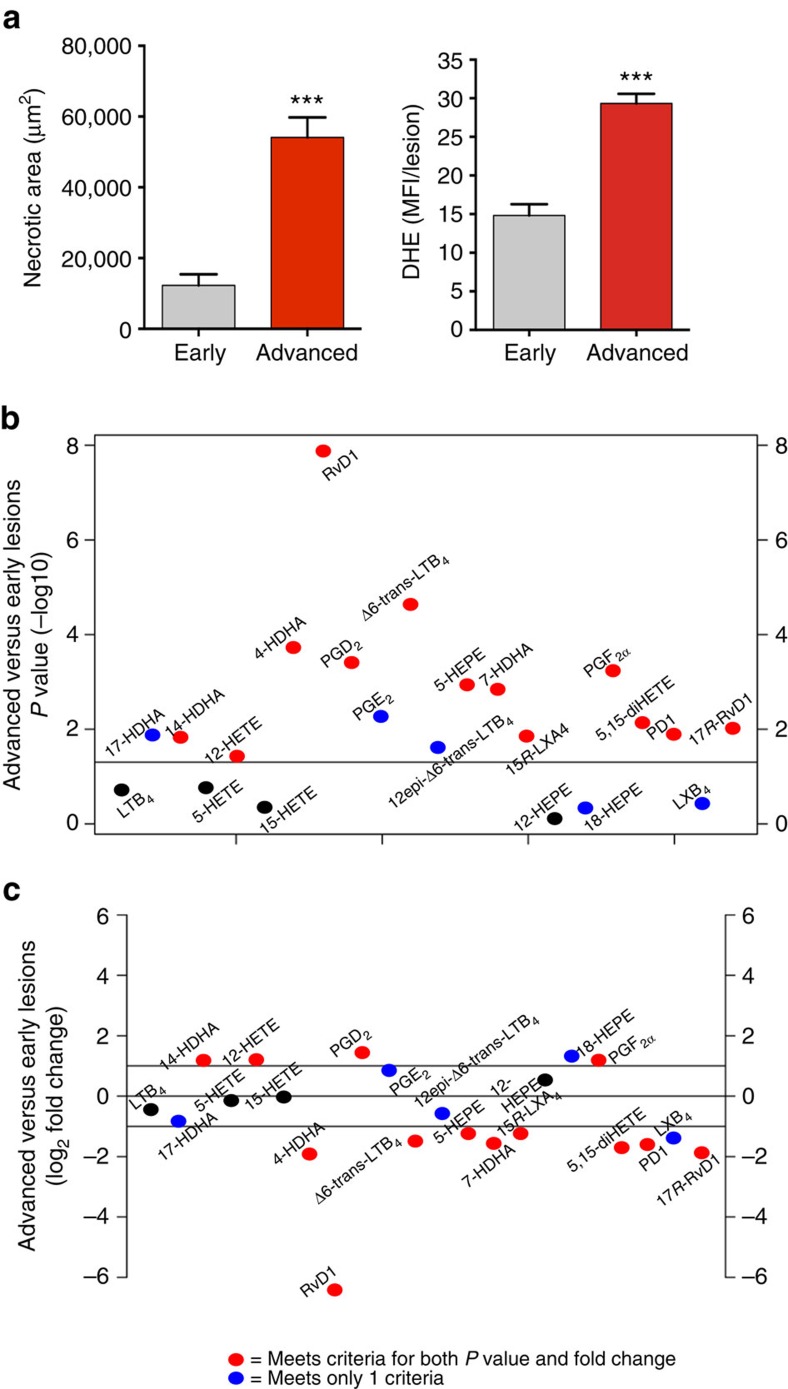
Advanced aortic root plaques of WD-fed *Ldlr*^*−/−*^ mice exhibit an imbalance in 5-LOX-derived SPMs and LTs compared with earlier stage lesions. (**a**) Aortic root lesions from *Ldlr*^*−/−*^ mice fed the WD for 8 weeks (Early) or 17 weeks (Advanced) were quantified for necrotic area and mean fluorescence intensity after DHE staining (data are shown as mean±s.e.m., *t*-test, ***P<0.001, *n*=7 for early lesions and *n*=11 for advanced plaques). (**b**,**c**) Analysis of lipid mediators in advanced versus early atherosclerotic plaques. Red dots indicate mediators that both reached statistical difference by Student's *t-*test (−log_10_ of the *P*-value) and changed by at least twofold (log_2_); blue dots indicate mediators that met only one of these criteria; and black dots represent lipid mediators that met neither of these criteria. For **b** and **c**, *n*=8 for early lesions and *n*=11 for advanced lesions.

To determine whether the findings with human plaques might be amenable to further experimentation in an animal model, we conducted targeted LC–MS/MS analysis of aortic arch and brachiocephalic artery (BCA) lesions of *Ldlr*^*−/−*^ mice fed a Western-type diet (WD) for 8 weeks (early lesions) or 17 weeks (advanced lesions). Similar to human vulnerable plaques, advanced murine plaques had significantly larger necrotic areas and more lesional OS compared with early lesions (Fig. 2a). We identified SPMs (RvD1, 17*R*-RvD1, 15*R*-LXA_4_ and PD1), SPM biosynthetic pathway markers (for example, 17-HDHA, 18-HEPE, 4-HDHA), and several pro-inflammatory lipid mediators, including LTB_4_ and prostaglandins (PGE_2_, PGD_2_ and PGF_2α_) (Fig. 2b; Supplementary Table 3). Using Metaboanalyst software (see ‘Methods' section), we found that several of these lipid mediators were significantly different in early versus advanced lesions (Fig. 2b). Among these changes, RvD1 emerged as being the most highly significantly changed mediator. To gain an appreciation for the magnitude and direction of these changes, we plotted the data as shown in Fig. 2c; red dots indicate those mediators above the twofold threshold that were significantly increased in advanced lesions, whereas red dots below the threshold were considered significantly decreased mediators in advanced lesions. The blue dots indicate mediators that were either significantly changed or changed by more than twofold, whereas the mediators indicated by black dots did not meet either criterion. The data show a striking ∼87-fold significant decrease in RvD1 in advanced versus early lesions. Other SPMs, such as 17*R*-RvD1, PD1 and LXB_4_, were also decreased in advanced lesions, although to a lesser extent than RvD1. In contrast, early and advanced lesions had similar amounts of LTB_4_ (Fig. 2c). Importantly, the levels of 15-PGDH were not different between Veh or RvD1-administered mice (Supplementary Fig. 4b), suggesting that the decrease of RvD1 in advanced plaques may be due to a production defect. Together, these data indicate that the progression of atherosclerosis in WD-fed *Ldlr*^*−/−*^ mice is associated with a marked decrease in RvD1, while pro-inflammatory LTB_4_ levels are maintained. These findings are quantitatively similar to those found in the comparison of vulnerable versus stable regions of human plaques.

### Administration of RvD1 suppresses plaque progression

**Figure 3:**
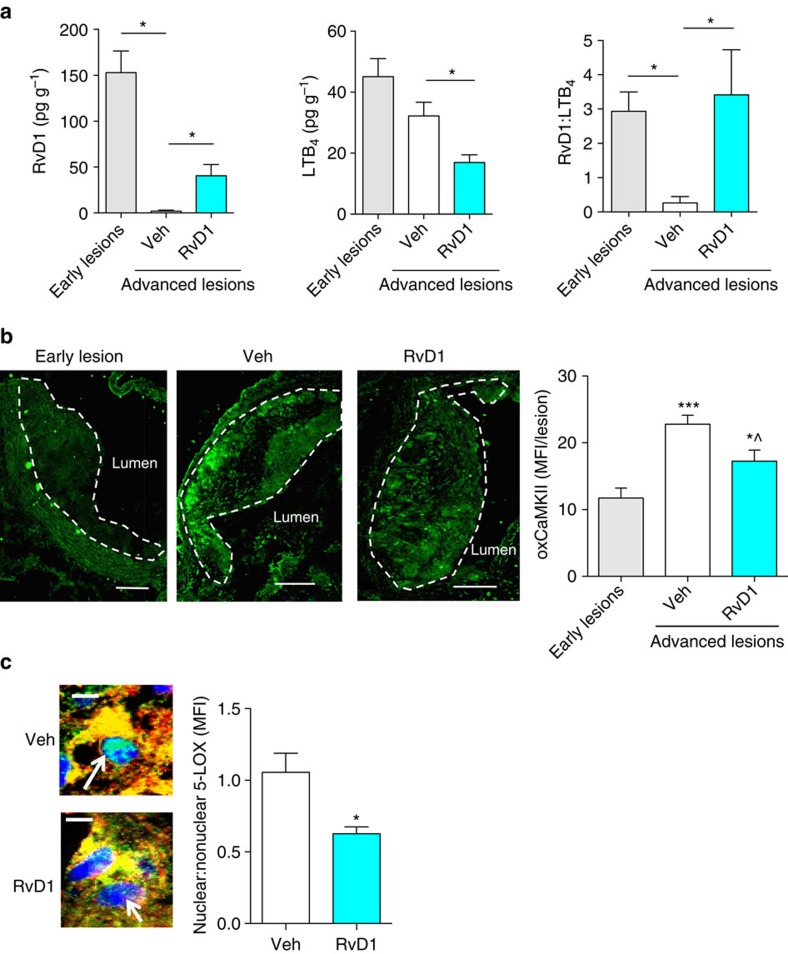
Administration of RvD1 to WD-fed *Ldlr*^−/−^ mice with established atherosclerosis restores lipid medicator balance. (**a**) Quantification of lesional RvD1 and LTB_4_ levels (left and middle panels) and RvD1:LTB_4_ ratio (right panel) in aortic lesions of 17-week WD-fed *Ldlr*^*−/−*^ mice that were administered vehicle control (Veh) or 100 ng RvD1/mouse (3 × /week i.p.) during weeks 12–17; the ratio data in 3a right panel are also shown for mice fed the WD for 8 weeks (Early lesions). For **a**, data are shown as mean±s.e.m., one-way ANOVA with Fisher's least significant difference *post-hoc* analysis, *P<0.05, *n*=8 for early lesions, *n*=11 for advanced lesions/Veh group, and *n*=10 for advanced lesions/RvD1 group. (**b**) Representative images and quantification of lesional oxCaMKII by immunofluorescence microscopic analysis. MFI data are shown as mean±s.e.m., one-way ANOVA with Tukey's multiple comparison test, *P<0.05 and ***P<0.001 early lesions versus other groups. ^*P*<0.05 Veh versus RvD1 groups, *n*=8 for early lesions, *n*=11 for advanced lesions/Veh group, and *n*=10 for advanced lesions/RvD1 group. Scale bar, 100 μm. (**c**) Representative confocal immunofluorescence images and quantification of 5-LOX localization in aortic lesional macrophages of 17-week WD-fed *Ldlr*^*−/−*^ mice given vehicle control (Veh) or RvD1 during weeks 12–17. Macrophage F4/80 is red, 5-LOX is green, and Hoechst (nuclei) is blue. Arrows indicate nuclear region. In these images, nuclear 5-LOX appears as blotchy light blue staining within the dark blue Hoechst-stained nuclei. The quantitative data are shown as mean±s.e.m., *t*-test, *P<0.05, *n*=11 for Veh group and *n*=10 for RvD1 group. Scale bar, 5 μm.

Because RvD1:LTB_4_ ratio was reduced in advanced plaques, we next questioned whether we could ‘restore' this ratio to the early lesional level by RvD1 administration to the mice and, if so, suppress advanced plaque progression. For these experiments, *Ldlr*^*−/−*^ mice were fed the WD for either 8 weeks (early lesions) or 17 weeks (advanced lesions), with vehicle control or 100 ng RvD1/mouse (3 × /wk i.p.) administered during the last 5 weeks of the advanced lesion cohorts. As expected, RvD1 administration increased lesional RvD1 levels (Fig. 3a, left panel). Importantly, the absolute level of RvD1 (40.6±12.2 pg g^−1^) was not supraphysiologic, as the level in early lesions was 152.9±23.6 pg g^−1^. Moreover, LTB_4_ levels in lesions were significantly decreased by RvD1 administration (Fig. 3a, middle panel), which is consistent with our previous finding that RvD1 lowers LTB_4_ in macrophages by increasing the ratio of non-nuclear:nuclear 5-LOX^16^. The overall net effect was that RvD1 treatment restored the advanced lesional ratio of RvD1:LTB_4_ to that of early lesions (Fig. 3a right panel). Moreover, other SPMs and pathway markers of SPM biosynthesis, including PD1, 17-HDHA, 15-HETE and MaR1 (ref. 23) were increased by RvD1 (Supplementary Fig. 5a; Supplementary Table 3). In contrast, the COX products PGE_2_, PGD_2_ and PGF_2α_ were not affected by RvD1 treatment (Supplementary Fig. 5b; Supplementary Table 3). Monohydroxy products of 5-LOX, including 5-HETE, 5-HEPE, 4-HDHA and 7-HDHA, were also detected and quantified (Supplementary Fig. 5c; Supplementary Table 3). The increase of 4-HDHA and the lack of suppression of the other monohydroxy 5-LOX products suggest that RvD1 does not act as a 5-LOX inhibitor. In summary, restoring RvD1 during the time period when earlier lesions are progressing to advanced lesions reestablishes the balance of SPM to pro-inflammatory leukotrienes.

On the basis of our previous data linking oxidative activation of CaMKII to an increase in nuclear 5-LOX and a decrease in the SPM:leukotriene ratio in cultured macrophages, we probed oxCaMKII in the early versus advanced murine lesions, including advanced lesions from mice subjected to RvD1 restoration. We observed that advanced lesions had significantly more immunoreactive oxCaMKII compared with early lesions and that RvD1 restoration significantly decreased lesional oxCaMKII in these lesions (Fig. 3b). Moreover, using confocal immunofluorescence microscopy, we observed that RvD1 restoration decreased the nuclear:non-nuclear 5-LOX ratio in advanced lesional macrophages (Fig. 3c; Supplementary Fig. 6). These combined data provide correlative *in vivo* support for the hypothesis that oxidative activation of CaMKII in advanced lesional macrophages contributes to an imbalance of 5-LOX-derived resolving versus inflammatory lipid mediators in a manner that can be prevented by RvD1 restoration.

**Figure 4:**
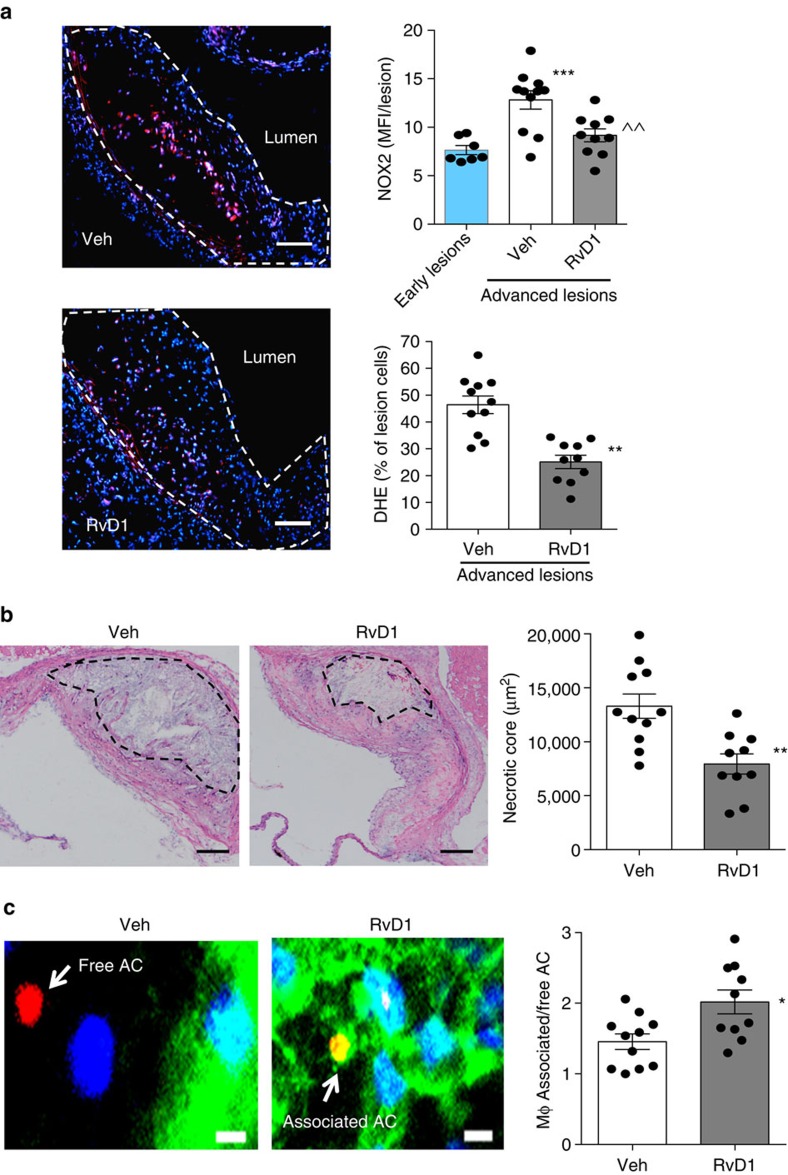
Administration of RvD1 to mice with established atherosclerosis suppresses lesional ROS and necrosis and enhances efferocytosis. (**a**) Representative images of DHE and quantification of immunoreactive NOX2 and DHE in aortic root lesions of 8-week and 17-week WD-fed *Ldlr*^*−/−*^ mice given vehicle control (Veh) or RvD1 for weeks 12–17 (Fig. 3). Scale bar, 100 μm. Data are shown as mean±s.e.m., one-way ANOVA with Tukey's multiple comparison test, ***P<0.001 early lesions versus other groups. ^^*P*<0.01 Veh versus RvD1 groups, *n*=8 for early lesions, *n*=11 for advanced lesions, *n*=10 for advanced lesions/RvD1 group. (**b**) Representative images and quantification of lesional necrosis in the two cohorts of mice. Scale bar, 100 μm. (**c**) Representative images and quantification of lesional efferocytosis, quantified as the ratio of TUNEL^+^ apoptotic cells (red) associated with lesional macrophages (green) versus apoptotic cells not associated with macrophages (‘free'). Yellow indicates red/green overlap, and blue indicates DAPI-stained nuclei. Scale bar, 5 μm. Data are shown as mean±s.e.m., *t*-test, *P<0.05, **P<0.01, *n*=11 for Veh group and *n*=10 for RvD1 group.

**Figure 5:**
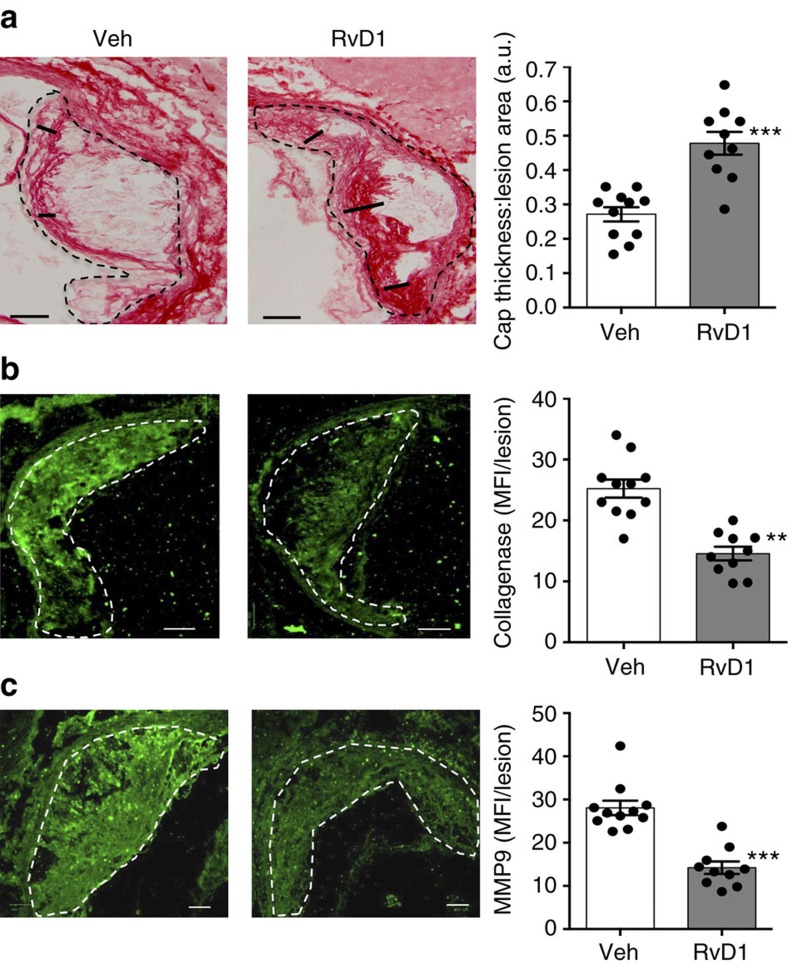
Administration of RvD1 to WD-fed *Ldlr*^−/−^ mice with established atherosclerosis enhances fibrous caps and decreases collagenase and MMP9. (**a**) Representative images and quantification of collagen of 17-week WD-fed *Ldlr*^*−/−*^ mice given vehicle control (Veh) or RvD1 for weeks 12–17 (Fig. 3). Scale bar, 100 μm. (**b**,**c**) Representative images and quantification of collagenase and MMP9. Scale bar, 100 μm. Data are shown as mean±s.e.m., *t*-test, **P<0.01 and ***P<0.001, *n*=11 for Veh group and *n*=10 for RvD1 group.

Having achieved the goal of restoring RvD1:leukotriene balance in advanced lesions, we then analyzed the mice for basic metabolic parameters and, most importantly, for hallmarks of advanced plaque progression that are associated with vulnerable plaques in humans^11^. RvD1 had no effect on body weight, plasma cholesterol, plasma insulin or plasma MCP-1 levels (Supplementary Fig. 7a–d). As expected, aortic root plaques of the vehicle-treated mice exhibited robust DHE and NOX2 staining, indicative of OS (Fig. 4a, top image and white bar), and large necrotic cores (Fig. 4b, left image and white bar of graph). We found that RvD1 markedly reduced both of these endpoints (Fig. 4a,b, grey bars of graph). Defective clearance of apoptotic cells (efferocytosis) in advanced plaques promotes plaque necrosis^2^,^12^, and, in other settings, RvD1 has been shown to enhance efferocytosis^24^. By quantifying macrophage-associated apoptotic cells versus free apoptotic cells in lesions as a measure of efferocytosis^25^, we found that RvD1 significantly enhanced lesional efferocytosis (Fig. 4c; Supplementary Fig. 8a) without affecting lesional apoptosis (Supplementary Fig. 8b). Another hallmark of advanced plaques is thinning of the ‘protective' fibrous collagenous cap that overlies necrotic areas^2^,^11^. While the aortic root lesions of the vehicle-treated control group had relatively thin fibrous caps, those in the RvD1 group had significantly thicker fibrous caps (Fig. 5a). Note that RvD1 did not affect lesional macrophage or smooth muscle cell content (Supplementary Fig. 7e,f), indicating that its protective effects were not being mediated by regulating the number of these cells in lesions. In this regard, advanced plaques exhibited robust staining of both collagenase and MMP9 (Fig. 5b,c), while RvD1 significantly reduced both endpoints (Fig. 5b,c). Together, these results indicate that RvD1 restores the RvD1:LTB_4_ balance in advanced plaques and, in association with this effect, suppresses key hallmarks of plaque progression.

## Discussion

The findings of this study provide critical molecular support to the concept that defective resolution of inflammation contributes to atherosclerosis progression^1^,^2^,^13^,^26^, and they offer a new framework to understand how this defect may develop. Moreover, the finding that advanced plaque progression can be suppressed by ‘normalizing' resolution:inflammation imbalance during a critical period of plaque transformation adds causative support for the concept and provides a mechanistic underpinning to recent studies showing the benefits of resolving mediator therapy in atherosclerosis^27^,^28^,^29^,^30^.

More than two decades ago, Serhan and colleagues^31^ found that angioplasty increased the levels of certain leukotrienes and lipoxins in human coronary arterial blood, suggesting that coronary arterial cells could carry out lipid mediator biosynthesis. Following that discovery, correlations were reported for high levels of plasma leukotrienes and plaque instability in humans^32^. In this regard, the LTB_4_ receptor BLT1 is expressed by human lesional cells and triggers inflammatory signalling pathways^33^. Leukotrienes promote chemotaxis of inflammatory cells and enhance the production of other pro-inflammatory mediators that, when in excess, can exacerbate vascular disease^34^. There have also been reports that atherosclerotic disease in humans is correlated with low levels of SPMs in the plasma, including aspirin-triggered LXA_4_ (refs 35, 36).

The situation is more complex with 5-LOX, which can give rise to either inflammatory or resolving mediators, and 12/15-LOX, which can promote either SPM synthesis or pro-atherogenic oxidation of LDL^1^,^26^,^37^,^38^,^39^. A possible determinant as to whether these pathways promote or suppress plaque progression may be the relative types of long-chain fatty acids in the diet, because essential omega-6 fatty acids (for example, AA) can promote the local synthesis of pro-inflammatory mediators, while omega-3 fatty acids can promote the local biosynthesis of pro-resolving mediators^5^. In *Apoe*^*−/−*^ mice, a high omega-3:omega-6 ratio was found to be associated with decreased atherosclerosis^40^, although whether this effect was caused by alterations in lipid mediators is not known. In humans, individuals with a specific *ALOX5* variant who also ingested a diet rich in AA had increased carotid atherosclerosis compared with individuals with non-variant *ALOX5*, while those with the same mutation but who ingested a diet rich in omega-3 fatty acids had less carotid atherosclerosis compared with the control population^41^. More recent work indicated a trend between EPA and a 5-LOX variant in lowering CVD risk (*P*=0.06)^36^. Further studies need to be carried out to determine the functions of these SNPs and to reconcile the results of a few clinical trials that failed to show a protective effect of dietary fish oils^42^. Other possible determinants of the balance of 5-LOX products include factors that affect the nuclear versus non-nuclear location of 5-LOX, such as oxysterols/OS as demonstrated here, and SPMs themselves, as recently published by us^16^. Other processes that can affect SPM production and that could, in theory, play roles in atherosclerosis include cell-cell communication and transcellular biosynthesis^5^; release of LX precursors from cellular phospholipids^43^; and action of 5-LOX activating protein (FLAP), which may play a role in LX production^44^,^45^,^46^,^47^. Leukotriene A_4_ hydrolase (LTA_4_H) is also emerging as an intriguing target for atherosclerosis, because inhibition of this enzyme has been linked to the decrease of leukotriene B_4_ and the increase of lipoxins^32^,^44^,^48^.

One of the most striking features of RvD1 administration was its ability to increase the fibrous cap. In support of these findings, RvE1 increased collagen in the context of periodontal ligament repair^49^ and 17*R*-RvD1 was shown to increase matrix synthesis in a model of arthritis^50^. On the other hand, RvD1 was found to decrease cardiac collagen in the setting of myocardial infarction^51^, and there are several reports that suggest SPMs exert anti-fibrotic actions^52^,^53^. Thus, the role of SPMs in fibrosis appears to be context-specific, and factors that control its actions in collagen synthesis versus degradation in advanced atherosclerosis represents an important area for future investigation.

Exogenously administered SPMs have shown benefit in other vascular settings, including injury-induced neointimal hyperplasia and monocyte recruitment during early atherogenesis^28^,^54^. However, these settings are very different from the one examined in this report—advanced atherosclerosis. Furthermore, mechanistic links represent a major gap in this area of research. Given the importance of defective efferocytosis in necrotic core formation and advanced plaque progression^2^, one key link likely involves the beneficial effect of SPMs on efferocytosis^5^,^55^, including that shown here for RvD1. Advanced plaques also have elevated OS^18^. In this context, we showed that 7-KC-induced OS in human macrophages lowered RvD1 levels and that DHE fluorescence in human lesions, which is an indicator of OS, showed a negative correlation with lesional RvD1. Pending more in-depth analysis of various ROS in the atherosclerotic lesions investigated in this study, our combined *in vitro* and *in vivo* data thus far suggest that OS may be one mechanism leading to lower RvD1 levels in advanced lesions. It is also possible that a deficiency in RvD1 exacerbates OS in advanced atherosclerosis, which could then contribute to defective efferocytosis^56^. In terms of therapeutic implications of these concepts, we showed recently that treatment of WD-fed *Ldlr*^−/−^ mice with plaque-targeted nanoparticles containing Ac2–26, a bioactive peptide derived from the pro-resolving protein called annexin A1, exhibited increased fibrous cap thickness and decreased OS and necrosis compared with control mice^27^. Ac2–26 and RvD1, the mediator used here, activate the same G-protein-coupled receptor called ALX/FPR2 (refs 24, 57). Thus, stimulating pro-resolving signalling via ALX/FPR2 may be a promising therapeutic strategy to prevent vulnerable plaque formation.

In summary, we have shown that the levels of 5-LOX-derived SPMs, particularly RvD1, are lower in human vulnerable versus stable plaques and murine advanced versus early lesions and that restoration of the RvD1:LTB_4_ ratio in murine plaques protects against advanced plaque progression. These findings add a critical new molecular link to the concept that resolution of inflammation is defective in advanced atherosclerosis and provide a rationale for developing ‘SPM-restoration' therapy to help prevent the clinical progression of atherosclerosis in humans.

## Methods

### Human carotid endarterectomy analysis

De-identified atherosclerotic plaques from elective endarterectomy procedures were obtained with informed consent and then immediately snap frozen in the operating room using liquid nitrogen. Specimens were then dissected into ‘vulnerable' and ‘stable' regions based on gross appearance. Gross necrotic regions were identified by dark brown regions and were dissected from less necrotic regions of the same donor. For human plaque histology, vulnerable and stable plaques from each donor were fixed using 10% formalin overnight at room temperature (RT). Tissue was then washed with PBS and decalcified using calcic-clear rapid solution (National Diagnostics) for 3 h at RT. Human plaques were placed in biopsy cassettes, processed in a Leica tissue-processing machine, and embedded in paraffin blocks. Sections were cut serially at 8-μm intervals and mounted on slides. Before staining, sections were deparaffinized in xylene and rehydrated in graded series of ethanol. For plaque necrosis measurements, the sections were stained with Harris' hematoxylin and eosin (H&E). Total lesional and necrotic areas were quantified using Image Pro Plus software. Lesional collagen was stained with picrosirius red as per the manufacturer's instructions (PolySciences), and the ratio of fibrous cap thickness to total lesion area was quantified with image processing software^27^,^58^,^59^. Dihydroethidium bromide (DHE, Life Technologies) was used per manufacturer's instructions, and total plaque mean fluorescence intensity (MFI) was quantified using ImageJ software. Vulnerable and stable lesions from each donor were then subjected to tandem mass spectrometry for lipid mediator analysis (see below for more details). Lipid mediators were calculated as picogram per gram of frozen tissue. The Columbia University IRB provided ethical approval for these studies and procedures were conducted in accordance with an approved IRB protocol.

### Human macrophage experiments

Buffy coats were purchased from the New York Blood Donor Center, and peripheral blood mononuclear cells (PBMCs) were isolated using Histopaque 1077 (Sigma). Briefly, buffy coat cells were carefully loaded onto Histopaque 1077 and then spun at 1,500 r.p.m. for 30 min at room temperature. The centrifuge was set to ‘no brake' to preserve the separation of the cells. PBMCs were collected and washed twice with PBS. Sixty million PBMCs were plated on a 10-cm petri dish for 30 min to allow for monocyte adhesion. After 30 min, non-adherent cells were removed by rinsing with PBS, and the attached cells were cultured with RMPI-1640 containing 10% FBS and 10 ngml^−1^ of recombinant human GM-CSF for 7 days^60^. (Fresh GM-CSF-containing media was supplemented on day 3). In some experiments, the macrophages (3 × 10^6^ cells/treatment) were incubated with 35 μM of 7KC or vehicle control for 5 h. The macrophages were then analyzed for ROS using a CellROX probe (Invitrogen) or fixed, permeabilized and stained with an antibody against cleaved caspase-3 (Cell Signaling). CellROX-stained images were acquired using a Bio-Rad Zoe fluorescence microscope, and images were analyzed using ImageJ software. Cleaved caspase-3 was quantified by flow cytometry using FlowJo software. In other experiments, the cells were sequentially incubated with 10 μM NAC for 1 h and then 35 μM of 7KC or vehicle for 5 h, after which either 10 μM DHA or vehicle was added for an additional 1 h. The media were then collected and assayed for RvD1 by ELISA (Cayman Chemicals).

### Macrophage 5-LOX localization

The ratio of nuclear to non-nuclear 5-LOX mean florescence intensity (MFI) was quantified by confocal immunofluorescence microscopy, as previously described^16^. Briefly, macrophages (60,000 cells per well in an 8-well coverslip) were fixed with 4% formalin for 15 min. After removal of the formalin, the cells were incubated with 1:100 anti-5-LOX antibody (Abcam #ab39347) in 1 × PermWash buffer (BD Biosciences) for 45 min and then with 1:200 goat-anti-rabbit IgG secondary antibody conjugated to Alexa 488 for 45 min. After counterstaining with Hoechst, the cells were imaged on a Leica confocal microscope. An average of 15–20 cells per treatment per donor were analyzed.

### Immunoprecipitation

Human macrophages were incubated for 3 h with 35 μM 7KC, 10 nM RvD1, 7KC+RvD1, or vehicle. For immunoprecipitation (i.p.) experiments, incubations were stopped using liquid nitrogen, and the cells were immediately collected in i.p. buffer. SureBeads (Bio-rad, #1614013) were conjugated to anti-CaMKII antibody (kind gift of Dr Harold Singer, Albany Medical College) or control IgG according to the manufacturer's instructions. The beads (100 μl) were added to 300 μl cell lysate, and the mixture was incubated overnight at 4 °C. The immunoprecipitates were collected by centrifugation and subjected to 10% SDS gel electrophoresis. Membranes were probed with anti-oxCaMKII at a 1:1,000 dilution (kind gift from Dr Mark E. Anderson, University of Iowa^22^,^61^), followed by an anti-rabbit light chain IgG (Abcam, #ab99697, 1:4,000).

### siRNA experiments

Human macrophages were incubated for 72 h with 1 μM of Accell human *Camk2g* siRNA SMARTpool (Dharmacon) or control siRNA (Dharmacon) in RPMI 1640 media containing 1% FBS. To confirm knockdown, cells were subjected to flow cytometry (below). The cells were then incubated for 5 h with 35 μM 7KC, 10 nM RvD1, 7KC+RvD1 (added at the same time), or vehicle control. 5-LOX localization and RvD1 ELISA were carried out as above.

### Flow cytometry

For signalling experiments, formalin-fixed human macrophages (above) were incubated with either anti-pMK2 (Cell Signaling, 3007) or anti-p-5-LOX-Ser271 (Cell Signaling, 3748) in 1 × permeabilization buffer (BD Biosciences) for 45 min on ice. For siRNA knockdown confirmation, macrophages (above) were fixed, then incubated with the CaMKIIγ antibody from Genetex in 1 × permeabilization buffer for 1 h on ice. Excess antibody was removed, and the cells were then incubated with goat-anti-rabbit IgG secondary antibody conjugated to Alexa 488 or Alexa 647 in 1 × permeabilization buffer for an additional 45 min on ice. Flow cytometric analysis was carried out using a FACsCaliber flow cytometer and FlowJo software.

### Murine atherosclerotic lesion analysis

Eight to ten week-old male *Ldlr*^*−/−*^ mice on the C57BL/6J background were purchased from Jackson Laboratory (stock # 002207) and placed on a WD (TD.88137, Harlan Talked) for 8–17 weeks. After 12 weeks on the WD, some of the cohorts were injected i.p. three times per week with 500 μl of sterile PBS (vehicle control) or RvD1 (100 ng/mouse) for an additional 5 weeks, while still on the WD. The Columbia University IACUC provided ethical approval for these studies and procedures were conducted in accordance with approved animal protocols. Necrotic core analysis was carried out on H&E-stained lesional cross sections as previously described^62^. Briefly, using light microscopy and image analysis software, boundary lines were drawn around areas lacking hematoxylin stained nuclei (acellular), and these areas were then quantified using a 10,000 μm^2^ threshold to avoid including regions that did not represent substantial areas of necrosis. Collagen staining was performed using picrosirius red as per the manufacturer's instructions (PolySciences) and analyzed as previously described^27^. All experiments were carried out in two separate cohorts each with ∼5 mice/group/cohort.

### Quantification of lipid mediators by metabololipidomics

Human endarterectomy or mouse atherosclerotic lesion samples were immediately snap frozen and stored at −80 °C. As described previously^63^, frozen samples were weighed, combined with 1 ml of ice-cold methanol containing deuterium-labeled internal standards (d_5_-RvD2, d_4_-LTB_4_, d_8_-5-HETE, d_4_-PGE_2_ and d_5_-LXA_4_), homogenized, and kept at −20 °C for 45 min to allow protein precipitation. Samples were centrifuged and supernatants were collected. Solid phase extraction was carried out on an automated extraction system (RapidTrace, Biotage) using C18 extraction cartridges. Following column conditioning with 3 ml of methanol followed by 6 ml of H_2_O, acidified samples (pH=3.5, HCl) were loaded onto the columns. Columns were washed with 4 ml of H_2_O followed by 5 ml of hexane, and lipid mediators were eluted with 9 ml of methyl formate. A steady stream of N_2_ gas was used to slowly concentrate the samples before complete re-suspension in methanol:water (50:50). Samples were injected and analyzed by LC–MS/MS using a high performance liquid chromatography system (HPLC; Shimadzu) coupled to a QTrap 6500 or QTrap5500 mass spectrometer (AB Sciex) operating in negative ionization mode using scheduled multiple reaction monitoring (MRM), information-dependent acquisition and enhanced product ion-scanning. A Poroshell column (100 mm × 4.6 mm × 2.7 μm, Agilent) was kept in a column oven maintained at 50 °C (ThermaSphere), and lipid mediators were eluted with a mobile phase consisting of methanol–water–acetic acid of 50:50:0.01 (vol/vol/vol) isocratic for 2 min that was ramped to 80:20:0.01 (vol/vol/vol) over 9 min, kept isocratic for 3.5 min, and then ramped to 98:2:0.01 (vol/vol/vol) for the next 0.1 min, which was maintained for 5.4 min at 0.5 ml min^−1^. Lipid mediators were identified using published criteria including retention time, MRM transitions, and diagnostic MS/MS ions and were quantified using standard curves for each mediator after normalization of extraction recovery based on internal deuterium-labelled standards^63^. To analyze the differences between early and advanced murine atherosclerotic lesions in an unbiased manner, we used Metaboanalyst 3.0 (http://www.metaboanalyst.ca). Missing values were imputed with half of the minimum positive value, and data were log transformed and autoscaled before analysis. Global changes between the groups were compared using Student's *t*-tests (*P*<0.05) and a fold-change threshold of 2.

### Immunofluorescence staining of murine lesions

For assessment of collagenase activity, aortic root lesions from Veh or RvD1-treated mice were analyzed using a collagenase assay kit from ThermoFisher in accordance with the company's guidelines. Briefly, collagenase substrate (1:50) was incubated with lesion sections overnight at 37 °C. After rinsing with PBS, the sections were imaged by fluorescence microscopy and analyzed using ImageJ software. For immunofluorescence microscopy, aortic root sections were fixed in ice-cold acetone for 5 min, washed in PBS, and blocked with DAKO protein block for 1 h at room temperature. Sections were then incubated overnight at 4 °C with antibodies against NOX2 (Abcam, ab80508), 15-PGDH (Genetex, GTX113740), MMP9 (Abcam, ab38898), oxCaMKII (Millipore, 07–1387), or alpha smooth muscle cell actin (Sigma, C6198), F4/80 (Bio-Rad, MCA497) all at 1:100 in the DAKO protein block solution. The sections were then rinsed, stained with a secondary antibody, rinsed again, viewed by fluorescence microscopy, and analyzed using ImageJ software.

### Murine aortic root confocal microscopy

Cross sections of murine aortic root lesion were stained at 4 °C with anti-5-LOX antibody and then anti-F4/80. The sections were counterstained with Hoechst to identify nuclei, viewed on a Nikon A1 confocal microscope, and analyzed using ImageJ software^16^.

### *In situ* efferocytosis

The efferocytosis assay was performed on frozen aortic root sections and was conducted using procedures previously described^25^. Briefly, an observer who was blinded to the identity of the samples quantified apoptotic (TUNEL-positive) nuclei (DAPI) that were associated with lesional macrophages, indicative of efferocytosis, or not associated with macrophages (‘free'). Macrophage-associated apoptotic cells followed the criteria of TUNEL-positive nuclei surrounded by or in contact with neighbouring F4/80+ macrophages. Free apoptotic cells exhibited nuclear condensation, loss of antibody F4/80 reactivity, and were not in contact with neighbouring macrophages.

### Statistical analysis

All mice were randomly selected to be in either the early or advanced group, and, for the advanced group, to receive either vehicle or RvD1. No mice were excluded from any of the groups. There were at least 10 mice per experimental group, and all data were found to fit a normal distribution. Results are shown as mean±s.e.m. For comparison between two experimental groups, the two-tailed Student's *t*-test was used, and for comparison among three experimental groups, one-way ANOVA with Fisher's least significant difference *post-hoc* analysis was used. The human macrophage experiments used at least three separate human donors per experiment, and so *n*≥3 for each experimental group. Results are shown as mean±s.e.m, and statistical differences were determined using the Kruskal–Wallis test with the Dunn's multiple comparison *post-hoc* analysis.

### Data availability

The data that support the findings of this study are available from the corresponding author upon reasonable request.

## Additional information

**How to cite this article:** Fredman, G. *et al*. An imbalance between specialized pro-resolving lipid mediators and pro-inflammatory leukotrienes promotes instability of atherosclerotic plaques. *Nat. Commun.* 7:12859 doi: 10.1038/ncomms12859 (2016).

## Supplementary Material

Supplementary Information Supplementary Figures 1-8 and Supplementary Tables 1-3.

## Figures and Tables

**Table 1:** Quantification of DHA-derived lipid mediators in stable and vulnerable regions of human carotid atherosclerotic plaques.

**Lipid mediator**	**Stable (pg g**^**−1**^ **(mean±s.e.m.))**	**Vulnerable (pg g**^**−1**^ **(mean±s.e.m.))**
		
*DHA-derived*		
4-HDHA	163.9±64.6	831.7±457.3
7-HDHA	49.2±15.9	144.9±48.0*
17-HDHA	71.4±10.4	129.5±19.1*
14-HDHA	303.1±67.2	839.4±280.3*
PD1	2.3±1.7	2.0±0.6
Mar1	ND	ND
RvD1	110.3±15.6	4.2±2.7*
RvD2	ND	ND
RvD3	ND	ND
RvD4	ND	ND
RvD5	ND	ND
RvD6	ND	ND
17*R*-RvD1	63.4±30.5	93.09±48.2
22-OH-PD1	ND	ND
13-HDHA	100.7±17.4	223.8±60.2*
17*R*-RvD3	1.8±1.4	0.4±0.1
4*S*,14*S*-diHDHA	ND	ND
Δ12-*trans*-MaR1	ND	ND
7-epi,Δ12-*trans*-MaR1	2.1±1.0	10.7±2.5*

DHA, docosahexaenoic acid; ND, not detected.

^*^P<0.05 (paired analysis).

**Table 2:** Quantification of AA- and eicosapentaenoic acid-derived lipid mediators in stable and vulnerable regions of human carotid atherosclerotic plaques.

**Lipid mediator**	**Stable (pg g**^**−1**^ **(mean±s.e.m.))**	**Vulnerable (pg g**^**−1**^ **(mean±s.e.m.))**
		
*AA-derived*		
5-HETE	1,660±309.5	4,389±1,490
12-HETE	3,782±817.2	6,759±1,959
15-HETE	1,571±227.9	1,894±326.3
5,15-diHETE	21.2±5.7	42.9±17
PGD_2_	164.8±42.5	335.8±192.7
PGE_2_	394.3±96.5	397.8±125.5
PGF_2α_	ND	ND
15*R*-LXA_4_	1,453±703.1	943±379.4
LTB_4_	596.3±133.1	810.5±184.8
		
*Eicosapentaenoic acid-derived*		
5-HEPE	107.2±18.1	294.6±69.6*
12-HEPE	411.4±127.8	1,449±685.7
15-HEPE	161±46.9	485.2±158.4*
18-HEPE	234.1±64.8	1,120±530
RvE1	ND	ND
RvE2	ND	ND
RvE3	ND	ND
5*S*,15*S*-diHEPE	ND	ND
LXA_5_	ND	ND
PGE_3_	ND	ND

AA, arachidonic acid; ND, not detected.

^*^P<0.05 (paired analysis).
