# Supplementary material for: An imbalance between specialized pro-resolving lipid mediators and pro-inflammatory leukotrienes promotes instability of atherosclerotic plaques
Source: Nat Commun. 2016 Sep 23;7:12859. doi: 10.1038/ncomms12859 (PMC5036151; doi:10.1038/ncomms12859)
Supplement: Supplementary Information — Supplementary Figures 1-8 and Supplementary Tables 1-3. [file ncomms12859-s1.pdf]

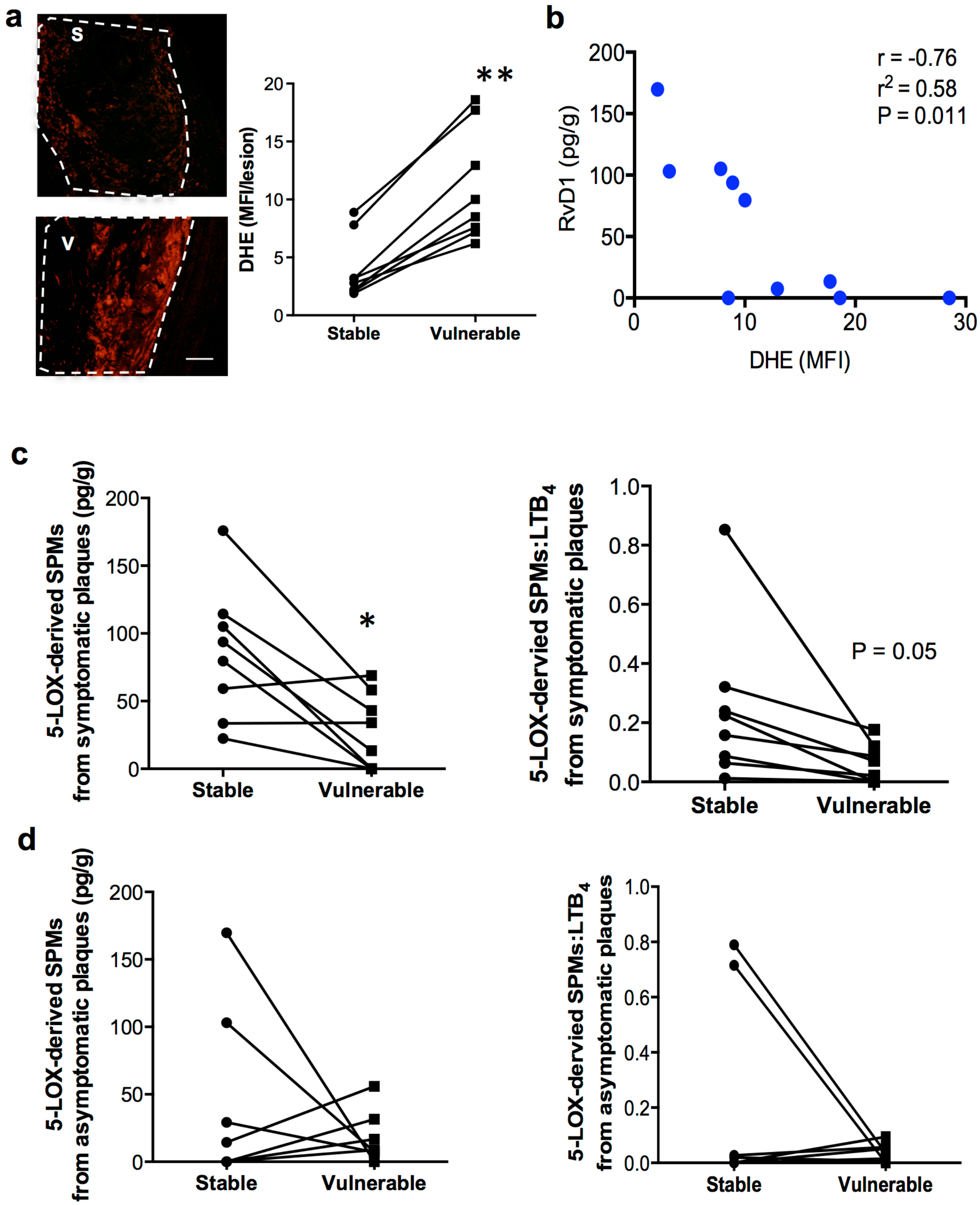

Supplementary Figure 1. Characterization of human carotid plaques. (**a**) Flash-frozen human plaques were separated into vulnerable (V) and stable (S), regions which were then quantified for mean fluorescence intensity (MFI) of DHE staining. *t*-test, ***P*<0.01, n = 8 donors; Scale bar, 100 μm. (**b**) The correlation of RvD1 and DHE levels in plaques. P = 0.011, n = 5 donors. (**c-d)** Comparison of 5-LOX-derived SPMs (RvD1 and LXA_4) and the SPM:LTB_4 ratio in vulnerable vs. stable regions from symptomatic plaques (**c**) vs. asymptomatic plaques (**d**). For (**c**) *t*-test, *P<0.05, n = 8 and for (**d**) n = 7 donors.

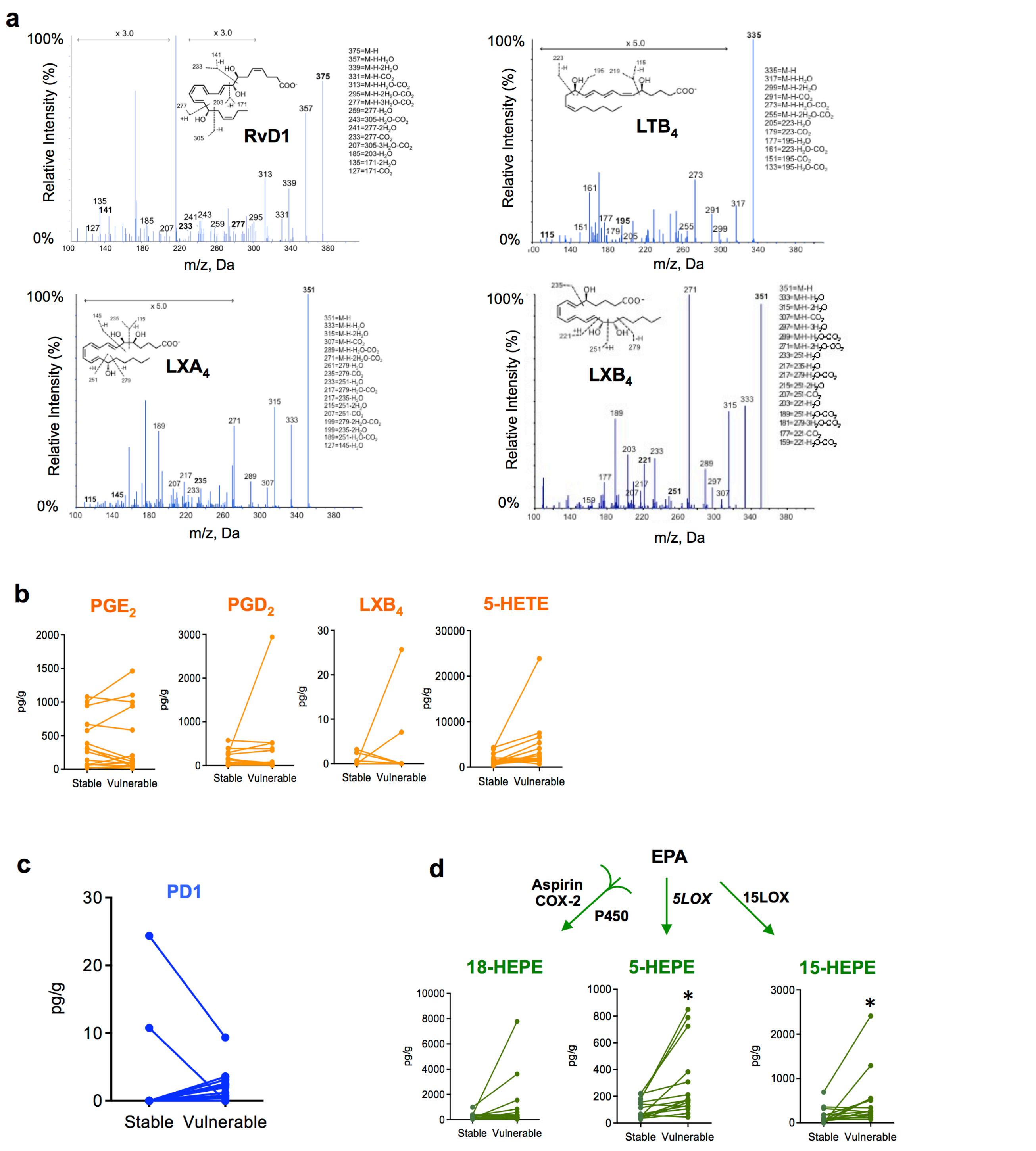

Supplementary Figure 2. Lipid mediators identified in stable and vulnerable regions of human carotid plaques. (**a**) Representative MS/MS spectra of RvD1, LTB_4, LXA_4, and LXB_4 identified in human atherosclerotic lesions. (**b-d**) Quantification of PGE_2, PGD_2, LXB_4 and 5-HETE; key eicosapentaenoic acid (EPA)-derived lipid mediators; and protectin D1 (PD1) in stable vs. vulnerable plaque regions (n = 15 donors). Data are shown as mean ± s.e.m., *t*-test, *$P<0.05$, n = 15 donors.

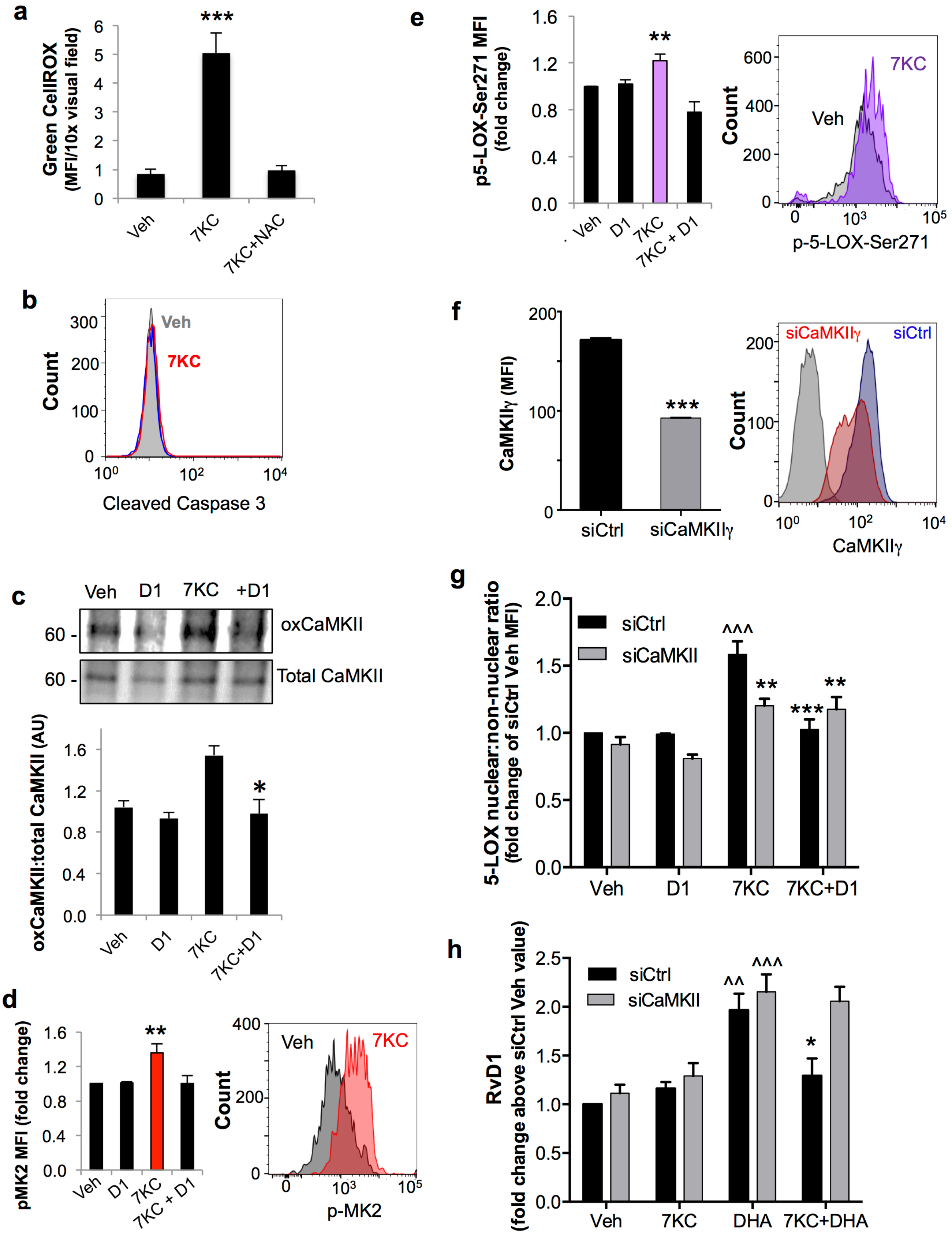

Supplementary Figure 3. Quantification of ROS, cleaved caspase 3, and 5-LOX signaling in human macrophages incubated with 7KC. (**a**) Human macrophages were pre-incubated with vehicle control (Veh) or 10 μM NAC for 1 h and then with Veh or 35 μM 7KC for 5 h. Reactive oxygen species were measured using the CellROX probe. Data are shown as mean ± s.e.m., Kruskal-Wallis

test, ****P*<0.001 vs. all other groups, n = 3 separate donors. (**b**) Human macrophages were incubated with vehicle or 35 μM of 7KC for 5 h and then monitored for cleaved caspase 3 by flow cytometry. Shown is a representative histogram of 1 of 3 separate donors. (**c**) Human macrophages were incubated with vehicle (Veh), 10 nM RvD1 (D1), 35 μM 7KC, or RvD1 + 7KC (7KC + D1) for 3 h. CaMKII was immunoprecipitated from whole cell lysates, and the immunoprecipitates were then probed for methionine-oxidized CaMKII (oxCaMKII) and total CaMKII by immunoblotting. A representative immunoblot is shown. Data shown are mean ± s.e.m., **P*<0.05, n = 3 individual experiments from 3 separate donors. (**d,e**) Quantification of p-MK2 and p-5-LOX-Ser271 in macrophages incubated for 3 h with vehicle, 35 μM 7KC, 10 nM RvD1, or 7KC + RvD1. Data shown are mean ± s.e.m., **P*<0.05, n = 3-4 separate donors; ns, non-significant. (**f**) Human macrophages were treated for 72 h with control siRNA (siCtrl) or *Camk2g* siRNA (siCaMKIIγ) and then subjected to flow cytometry to confirm knockdown. The left panel shows quantification of the flow data (mean ± s.e.m., ****P*<0.001, n = 3 separate donors). The right panel is a representative flow cytometry histogram where gray is the IgG group, red is the siCaMKIIγ group, and blue are siCtrl group. (**g**) Human macrophages were treated for 72 h with control siRNA (siCtrl) or *Camk2g* siRNA (siCaMKII) and then incubated for 5 h with Veh, RvD1, 7KC, or 7KC + RvD1. Incubations were stopped with 4% formalin, and cells were permeabilized and subjected to anti-5-LOX immunofluorescence confocal microscopy. Images were acquired on a Leica confocal microscope, and nuclear:non-nuclear 5-LOX MFI was quantified using Image J. Data are shown as mean ± s.e.m. (n = 4 separate donors). Statistical analysis was conducted using two-way ANOVA with Tukey multiple comparison test (***P*<0.01, ***P<0.001 for siCtrl 7KC *vs*. other treatments; ^^^P<0.001 for siCtrl Veh *vs*. siCtrl 7KC). (**h**) Human macrophages were incubated as in Fig 1e, and the media were assayed for RvD1 by ELISA. Data are shown as mean ± s.e.m. (n = 5 separate donors). Statistical analysis was conducted using two-way ANOVA with Tukey multiple comparison test (**P*<0.01 for DHA vs. siCtrl 7KC+DHA; ^^P<0.01, ^^^ P<0.001 for siCtrl Veh vs. other treatments).

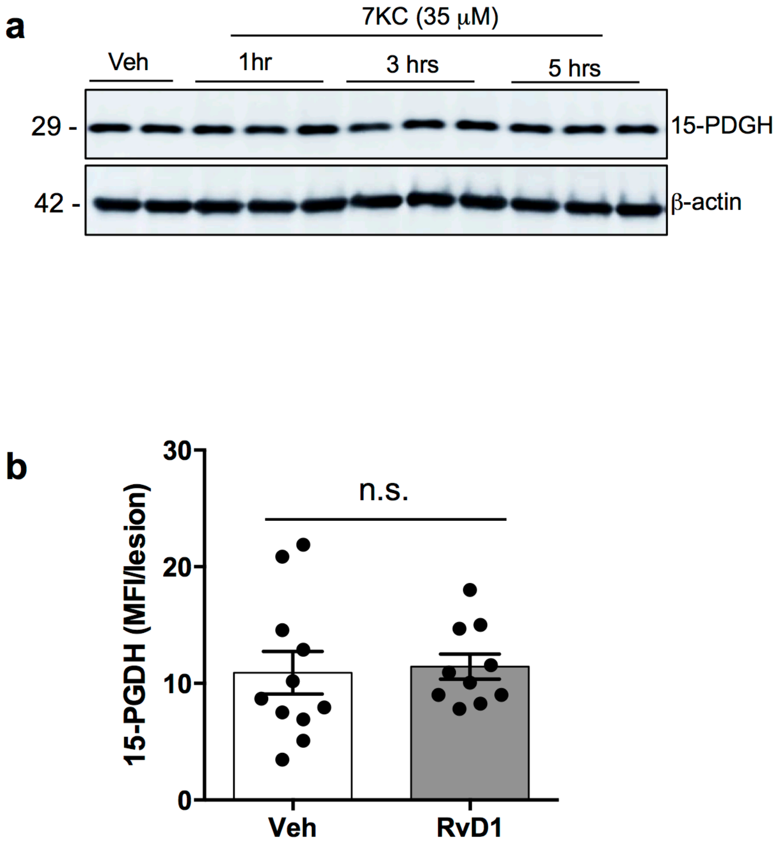

Supplementary Figure 4. 15-PGDH levels in human macrophages and murine lesions. (**a**) Human macrophages were incubated with 35 μM 7KC for 1, 3, or 5 h, and then cell lysates were subjected to immunoblot analysis for 15-PDGH and β-actin. Shown is a representative immunoblots image from one of two separate donors, with each time point carried out in triplicate. (**b**) Aortic root lesions of 17-week WD-fed *Ldlr*$^{-/-}$ mice treated with vehicle control (Veh) or RvD1 for weeks 12-17 (see Fig. 3) were quantified for 15-PGDH MFI by immunofluorescence microscopy. Data are shown as mean ± s.e.m., *t*-test, n.s, not significant, n = 11 for Veh group and n = 10 for RvD1 group.

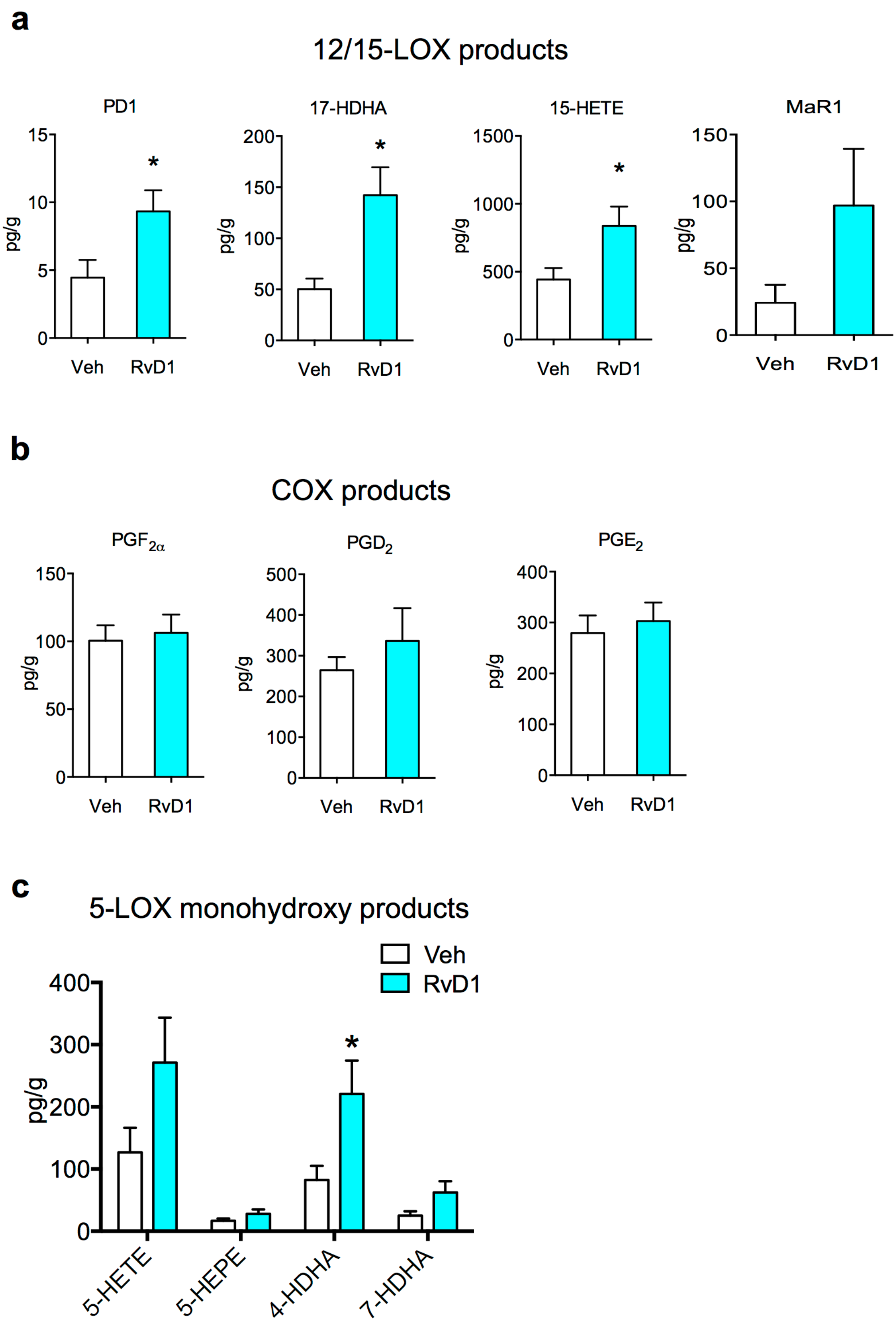

Supplementary Figure 5. Lesional lipid mediators after administration of RvD1 to WD-fed *Ldlr*$^{-/-}$ mice with established atherosclerosis. (**a-c**) Aortic root lesions of 17-week WD-fed *Ldlr*$^{-/-}$ mice given vehicle control (Veh) or RvD1 for weeks 12-17 (see Fig. 3) were quantified for the 12- and 15-LOX-derived products protectin D1 (PD1), 17-HDHA, 15-HETE, and maresin 1 (MaR1); cyclooxygenase-derived prostaglandins $PGF_{2\alpha}$, PGE_2, and PGD_2; and the 5-LOX-generated monohydroxy products 5-HETE, 5-HEPE, 4-HDHA, and 7-HDHA. Data are shown as mean ± s.e.m., *t*-test, *$P<0.05$, n = 11 for Veh group and n = 10 for RvD1 group.

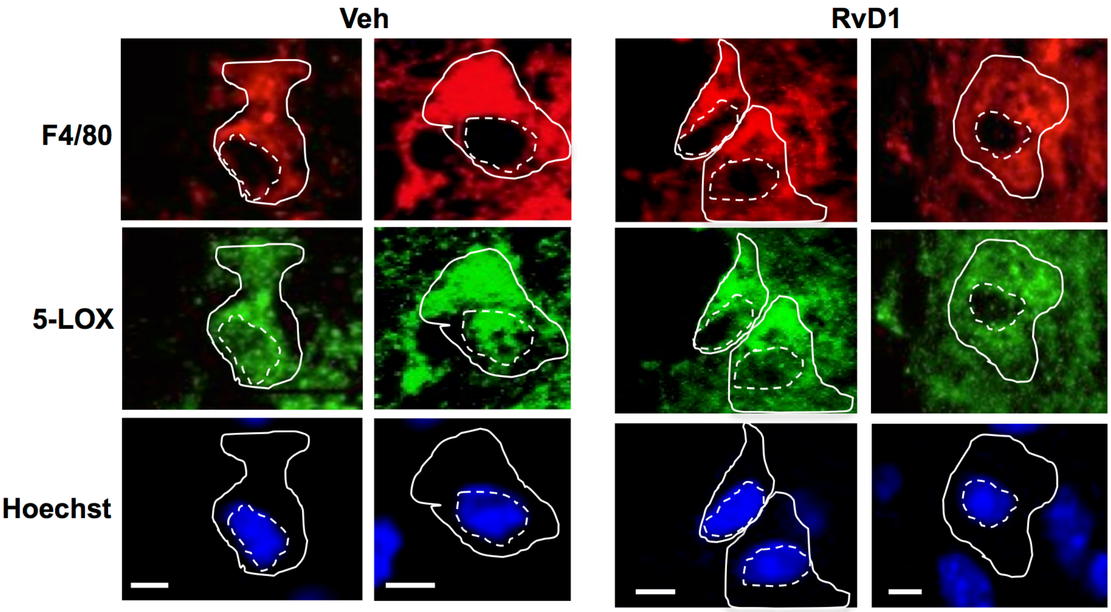

Supplementary Figure 6. Gallery of nuclear 5-LOX in aortic lesional macrophages. Aortic root sections were sequentially incubated with anti-5-LOX antibody overnight at 4°C followed by anti-F4/80 for 2 h at room temperature. Sections were counterstained with Hoechst and analyzed using a Nikon A1 confocal microscope and ImageJ software. Images display F4/80 in red, 5-LOX in green, and Hoechst in blue. Nuclei are outlined by a hashed line, and individual macrophages are outlined by a solid line. Scale bar, 5 μm.

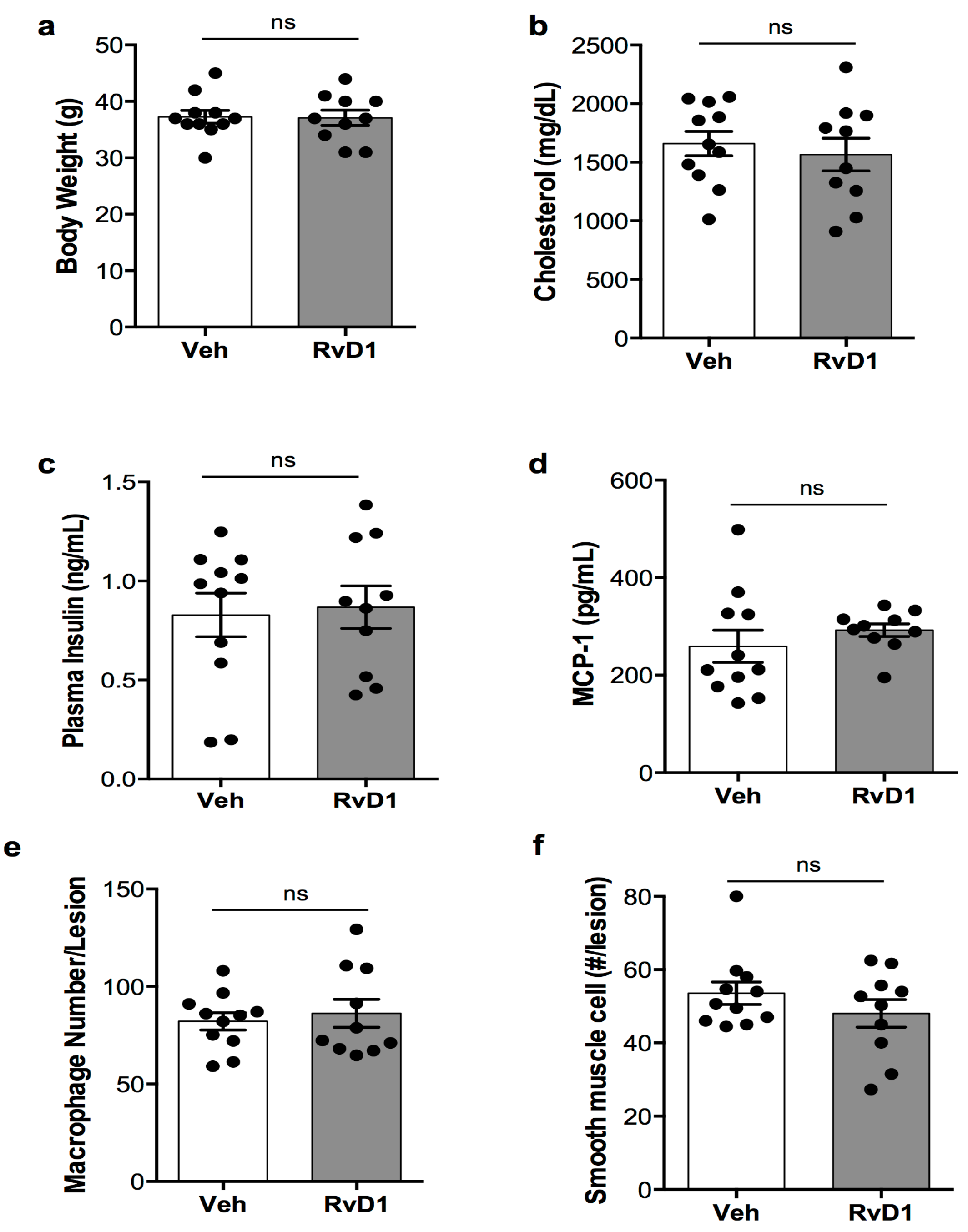

Supplementary Figure 7. Systemic and metabolic parameters and local cell counts in *Ldlr*$^{-/-}$ mice treated with Veh or RvD1. (**a-c**) Body weight, plasma cholesterol levels, and plasma insulin levels, in 17-week WD-fed *Ldlr*$^{-/-}$ mice given vehicle control (Veh) or RvD1 for weeks 12-17 (see Fig. 3). (**d**) Plasma levels of MCP-1 in Veh vs. RvD1 groups were analyzed by ELISA. (**e,f**) Macrophage and smooth muscle cells numbers in lesions were quantified by immunofluorescence and image processing. Data are shown as mean ± s.e.m., *t*-test, n.s., not significant, n = 11 for Veh group and n = 10 for RvD1 group.

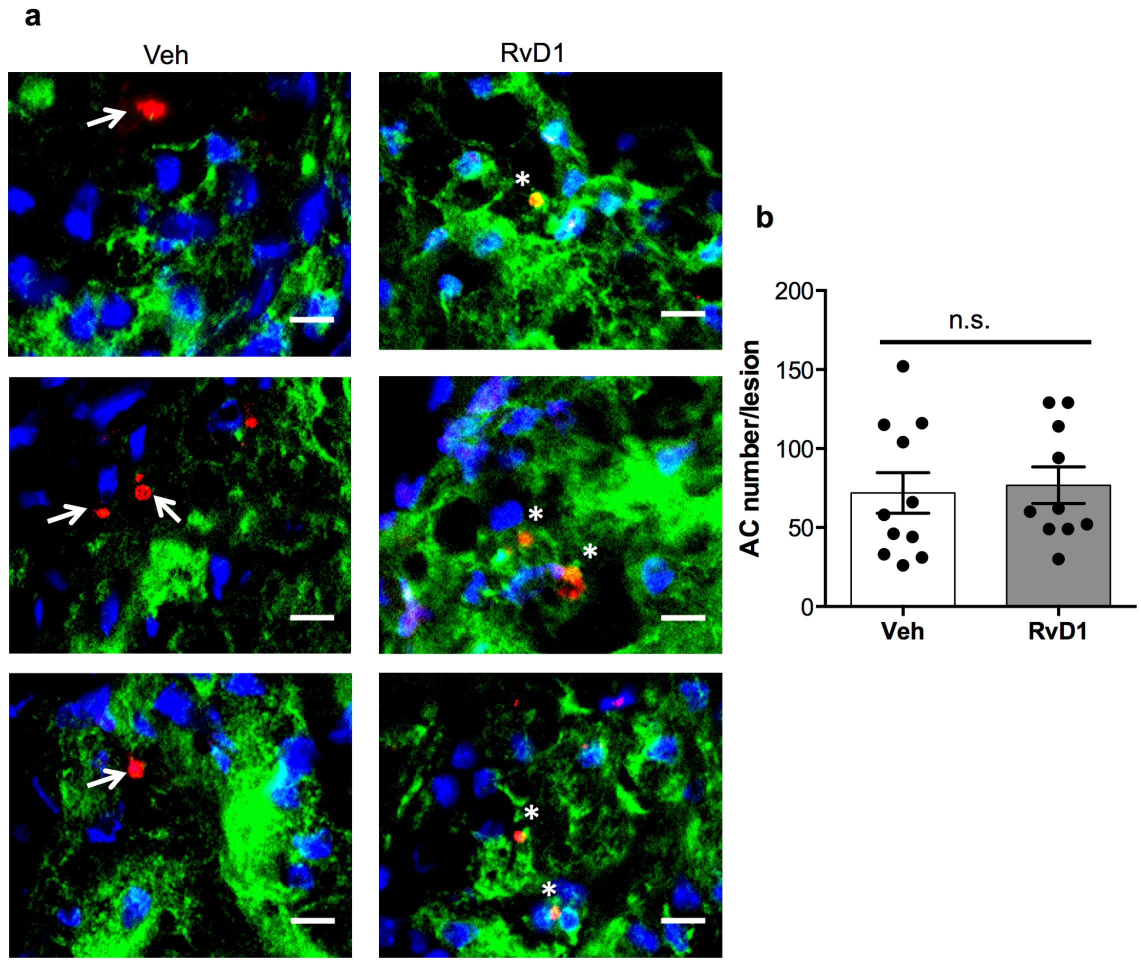

Supplementary Figure 8. Gallery of images from in-situ efferocytosis analysis of lesions of *Ldlr*$^{-/-}$ mice treated with Veh or RvD1. (**a**) In situ efferocytosis analysis, as described in Supplementary Methods, was conducted on aortic root sections from 17-week WD-fed *Ldlr*$^{-/-}$ mice given vehicle control (Veh) or RvD1 for weeks 12-17 (see Fig. 3). The macrophage marker F4/80 is shown in green, apoptotic cells (ACs) are shown in red, and nuclei are shown in blue. White arrows indicate free apoptotic cells, and white asterisks indicate associated ACs. Scale bar, 10 μm. (**b**) Quantification of total ACs per lesion. Data are shown as mean ± s.e.m., n.s., not significant, n = 11 for Veh group and n = 10 for RvD1 group.

Supplementary Table 1. Clinical characteristics of patients who underwent an elective endarterectomy.

Demographics	
Age, yr (mean ± SEM)	69 ± 2.4
Male	13/15
Female	2/15
Anthropometrics	
Body weight, kg (mean ± SEM)	87 ± 4.2
Height, m (mean ± SEM)	1.8 ± 0.02
BMI (mean ± SEM)	28 ± 1.1
CVD risk factors and clinical classification of carotid lesions	
Smoking	3/15
Diabetes	5/15
Asymptomatic	7/15
Symptomatic	8/15
Aspirin and Statin use	
Aspirin	15/15
Statin	12/15

Supplementary Table 2. Human plaque and mouse aorta specimen weights used for LC-MS/MS.

Human plaques (g)	**Stable (n=15)**	**Vulnerable (n=15)**
	0.08 ± 0.01	0.42 ± 0.06
Mouse aorta (mg)	**Early (n=8)**	**Advanced (n=11)**
	9.41 ± 0.57	14.10 ± 1.03

Data are mean ± s.e.m.

Supplementary Table 3. Quantification of docosahexaenoic acid-derived lipid mediators in aortic lesions of *Ldlr*$^{-/-}$ mice fed the WD for 8 weeks (Early Lesions) or 17 weeks (Advanced Lesions), with either Vehicle or RvD1 administered during weeks 13-17.

**Lipid Mediator**	**Early Lesion**	**Advanced Lesion-Vehicle**	**Advanced Lesion-RvD1**
	pg/g (mean ± SEM)	pg/g (mean ± SEM)	pg/g (mean ± SEM)
**Docosahexaenoic acid-derived**			
4-HDHA	317.3 ± 51.6	82.6 ± 22.7*	220.9 ± 53.6§
7-HDHA	76.3 ± 10.2	25.2 ± 7.1*	62.4 ± 18.0
17-HDHA	91.0 ± 9.2	50.2 ± 10.3*	142.2 ± 27.3§
14-HDHA	65.6 ± 9.1	141.4 ± 25.8*	152.4 ± 28.8
PD1	13.9 ± 3.5	4.5 ± 1.3*	9.3 ± 1.5§
Mar1	48.7 ± 31.9	24.2 ± 13.5	96.9 ± 42.4
RvD1	152.9 ± 23.6	1.8 ± 1.2*	40.6 ± 12.2§
RvD2	ND	ND	ND
RvD3	ND	ND	ND
RvD4	ND	ND	ND
RvD5	ND	ND	ND
RvD6	ND	ND	ND
17*R*-RvD1	1028.0 ± 96.7	275.0 ± 86.1*	335.4 ± 101.8
22-OH-PD1	ND	ND	ND
13-HDHA	417.9 ± 47.3	122.8 ± 26.9*	286.2 ± 68.1§
17*R*-RvD3	ND	ND	ND
4*S*,14*S*-diHDHA	ND	ND	ND
Δ12-*trans*-MaR1	ND	ND	ND
7-epi,Δ12-*trans*-MaR1	ND	ND	ND

*= P< 0.05 (Early vs. Advanced Lesion-Vehicle); § = P< 0.05 (Advanced Lesion-Vehicle *vs*. Advanced Lesion-RvD1). ND stands for not detected.

Supplementary Table 3 cont. Quantification of Arachidonic acid-derived lipid mediators in aortic lesions of *Ldlr*$^{-/-}$ mice fed the WD for 8 weeks (Early Lesions) or 17 weeks (Advanced Lesions), with either Vehicle or RvD1 administered during weeks 13-17.

**Lipid Mediator**	**Early Lesion**	**Advanced Lesion-Vehicle**	**Advanced Lesion-RvD1**
	pg/g (mean ± SEM)	pg/g (mean ± SEM)	pg/g (mean ± SEM)
**Arachidonic acid-derived**			
5-HETE	138.1 ± 8.4	126.7 ± 39.8	271.1 ± 72.3
12-HETE	1173.0 ± 111.3	2699.0 ± 572.9*	3334.0 ± 536.2
15-HETE	451.1 ± 43.6	442.7 ± 84.7	837.4 ± 142.5§
5,15-diHETE	1225.0 ± 267.8	381.9 ± 144.4*	665.4 ± 333.2
PGD_2	100.7 ± 21.4	264.5 ± 32.2*	336.5 ± 80.3
PGE_2	152.3 ± 19.6	279.4 ± 34.5*	303.0 ± 36.4
$PGF_{2\alpha}$	45.2 ± 4.9	100.5 ± 11.3*	106.3 ± 13.4
15*R*-LXA_4	164.2 ± 32.4	68.3 ± 25.2*	100.5 ± 34.9
LTB_4	45.1 ± 5.9	32.2 ± 4.5	16.9 ± 2.6§
Δ6-*trans*-LTB_4	23.5 ± 1.8	8.4 ± 1.3*	28.1 ± 7.1§
12epi-Δ6-*trans*-LTB_4	10.3 ± 1.1	6.8 ± 1.7	20.3 ± 3.8§
20-OH-LTB_4	ND	ND	ND
20-COOH-LTB_4	ND	ND	ND
11-HETE	564.3 ± 45.0	716.3 ± 90.3	1252.0 ± 254.9
5*S*-6*R*-diHETE	ND	ND	ND
LXB_4	122.2 ± 28.4	39.6 ± 13.7*	80.5 ± 28.6
LXA_4	ND	ND	ND

*= P< 0.05 (Early vs. Advanced Lesion-Vehicle); § = P< 0.05 (Advanced Lesion-Vehicle *vs*. Advanced Lesion-RvD1). ND stands for not detected.

Supplementary Table 3 cont. Quantification of Eicosapentaenoic acid- acid-derived lipid mediators in aortic lesions of *Ldlr*$^{-/-}$ mice fed the WD for 8 weeks (Early Lesions) or 17 weeks (Advanced Lesions), with either Vehicle or RvD1 administered during weeks 13-17.

Lipid Mediator	Early Lesion	Advanced Lesion-Vehicle	Advanced Lesion-RvD1
	pg/g (mean ± SEM)	pg/g (mean ± SEM)	pg/g (mean ± SEM)
**Eicosapentaenoic Acid derived**			
5-HEPE	40.7 ± 4.6	16.9 ± 3.7*	28.2 ± 6.9
12-HEPE	432.7 ± 41.7	614.7 ± 149.3	631.4 ± 107.6
18-HEPE	15.9 ± 3.7	39.2 ± 24.3	44.42 ± 14.7
RvE1	ND	ND	ND
RvE2	ND	ND	ND
RvE3	ND	ND	ND
5*S*,15*S*-diHEPE	ND	ND	ND
LXA_5	ND	ND	ND
PGE_3	ND	ND	ND

*= P< 0.05 (Early vs. Advanced Lesion-Vehicle); § = P< 0.05 (Advanced Lesion-Vehicle vs. Advanced Lesion-RvD1). ND stands for not detected.
